# Proceedings of the Nineteenth Annual Meeting of the Northern Ohio Dental Association

**Published:** 1878-08

**Authors:** 


### PROCEEDINGS OF THE NINETEENTH ANNUAL MEETING OF THE NORTHERN OHIO DENTAL ASSOCIATION.

ELYRIA, OHIO, MAY 14, 1878—MORNING SESSION.

Meeting called to order at ten o'clock in the rooms of Dr. Kelsey, by J. W. Lyder, President.

The minutes of the last annual meeting were read and approved.

On motion of Dr. Buffett the chair was instructed to fill any vacancies in offices and committees.

The chair then appointed Drs. Knowlton, and Kelsey to act with Dr. Buffet on the board of examiners, and Dr. Merritt to act with Dr. Buffet on the executive committee.

On motion professional visitors, including physicians, were invited to take part in the deliberations of the Association.

The executive committee then presented the following subjects for discussion:

1. Professional duties of the dentist.

2. Necrosis and exfoliation of the alveolar processes; causes and treatment.

3. Chronic inflammation and tumefaction of the gums, attended by recession of their margins from the necks of the teeth; causes and treatment.

4. Materials for and methods of filling teeth.

The first topic was opened by Dr. Kelsey, of Elyria, who read a short paper on the subject.

Dr. Buffet, of Cleveland, followed in a spirited manner. He said, I speak always with embarrassment, but we owe it as a duty to our brothers to speak here to-day if no where else. Life is full of duty—duty to our professional brothers, to our patients, to society, to our families. The duty of the dentist is no greater, however, than that of any professional man. Every man who enters any profession takes upon himself a special duty. He says to the world, trust me; I am qualified to do the work in which I am engaged. And society expects and demands that he shall fulfill his obligations. The first thing of importance for us then is to qualify ourselves for our trusts and place ourselves in a position for the highest qualification. Dentists are brought in contact with all classes; they are called upon to deal with deformities, with degeneracy and decay. Their skill is required to combat the entailed evils of sinful indulgences. Theirs is a labor of mercy, bringing relief to suffering humanity. And no one who is not thoroughly qualified, who is not the master of his profession is fit for the high calling of the dentist. The mere extracting and filling of teeth is not enough. Indeed, I hope that in the near future the wholesale and reckless slaughtering of teeth, which was a characteristic of the past, will be done away with. There must be advancement in knowledge, improvements and in methods. We owe it to the profession and to society to thoroughly qualify ourselves for our labors. This duty is closely allied to that of the physician. If he holds himself out to society as capable of answering the demands to be made upon him, he is held to a rigid fulfillment of his profession of competency, and if he is found to be unqualified, he is looked upon with scorn, while he who meets the requirements is given a place of honor and esteem. In the same way does the community measure the worth of the dentist, and he is held to the same rigid compliance with the duties devolving upon him. Not that we can all reach the goal of perfection; none can; but we can all strive to attain higher ground, and when we find an earnest and sincere worker we can and should extend the cordial hand of sympathy and assistance. Here is the place for professional sympathy and aid. Promises avail nothing here. If we go to our offices with the spirit of sharp, carping criticism reflecting unkindly and unjustly upon the labors of our brothers we are unprofessional. To be sure some of the best operators are unprofessional, Some of them are dishonest, untruthful in their representations, uncharitable in their criticisms. But this is not right. Honesty and sincerity should be at the bottom of all our intercourse and dealings, and especially should it be so in all our connections with our professional brethren. It is not unfrequent for a patient to leave the care of one dentist and present himself for treatment to another. How apt is he to use words or a manner at least that reflects discredit upon his predecessor, indicating that the patient has not been properly dealt with. It is unkind, it is unprofessional; it does not give a patient a very high estimation of the profession. The occupation of dentistry was at one time a reproach and the occasion of slurs, which came from the physician. A dentist was called a cut throat, a fraud, etc. Dentists were not and are not, as a class, all they should be, but they gave us far more than we deserved. We recognised the value and necessity of instruction. Dentistry consists simply in filling and extracting teeth. We must have instruction and we must look to those whose knowledge and experience qualify them to lead us to the true ends of the profession. We must have schools and professors where and of whom this knowledge and qualification can be gained. The taking of degrees has little to do with it. As Robert Burns says, a man 's a man for a' that," but we must have the proper qualifications and the proper evidences of it. The subject of uniformity in prices I do not think should be made very prominent in our discussions. It is possible here in smaller towns and villages to make prices uniform, but in the city it is different. It does not follow that because a man has less for his work than some one else that he is unprofessional. A yonng man who is well qualified should not be asked to come up to the standard of an old practitioner who has a busiuess established and a reputation made. The younger members of the profession should have the privilege of establishing a lower fee when making a start, and not be regarded as unprofessional in so doing. He should be able to say in honesty and candor, "I do this work at this price to earn my living." The young men must live, and they must have business to keep them alive. People are willing to pay a good price to you and me who have experience and a reputation. But it takes time to secure these, and one of less experience and reputation can not command as high a price. But if he claims to charge an established price and then cuts the price, that would be decidedly unprofessional. In all professions the nature of the man stands out in time, and the world judges him as he is, and if he is found to be worthy of confidence, his business and his prices will both advance, and the latter be cheerfully paid,

I hear the question asked "What do you say to the manufacture of cheap teeth—say ten dollars per set?" I say that if it is done honestly and without concealment, I think it is perfectly legitimate; but if it is done under the pretense of turning out first class work, it is entirely unprofessional. If one undertakes to do work for that price he can not do good work, and if he pretends that he can, his patients will all be pricked by the porcupine quills that stick out of the man. He may for a time draw custom from his neighbor, but in the end he will lose, and not the professional man. If one can not afford to pay for better and you have time, I think it perfectly legitimate to do cheap work. You cannot let a patient suffer because he can not pay for your best work, any more than the physician who is called upon to administer to a poor man. No man with a Christian sentiment would refuse to assist a sufferer. We are to relieve humanity not to labor for the accumulation of wealth entirely. To refuse to aid those who come to us in bodily anguish, because they are too poor to pay, would be like the New York divine who sent the dead body of the poor actor to the little church around the corner for burial. That would be the most unprofessional thing we could do.

Dr. Stroud, of Sandusky, followed. He endorsed the views of the gentleman who preceded him. He had lived in Sandusky fourteen years, and had seen many ups and downs in dentistry, and almost invariably the members of the profession who cut established prices were the ones who suffered in the end. He found that in the experience of every practitioner were many poor people. He had done a considerable gratuitous practice in giving them relief, and he thought it perfectly legitimate to use such material in his work as his patients could afford to pay for.

Then followed a few scattering remarks, when the meeting adjourned.

AFTERNOON SESSION.

The Association met in the parlor of the Beebe House, at the request of B. F. Turner, proprietor. The minutes of the morning meeting were read.

J. F. Siddall, of Oberlin, read a spicy paper upon the improvements in dentistry, comparing the meager and insufficient information of thirty and fourty years ago with the vast fund of positive knowledge and the improved methods of today. He thought the condition of dentists had improved financially; that they were more at liberty to seek their own diversion. In fact, if he was square with the world he could lay down his forceps and go a fishing, and who should say nay.

Dr. Chandler, of Sandusky, disagreed with Dr. Siddall; he did not think that a man, if straight with the world, could at will close his office and seek recreation. The true dentist with sympathy ever going out to his suffering friends, and quick desire for the mitigation of all the evils that lie within the reaeh of his skill, will always be at his post and in the harness. And he will be honest, patient and kind to his patients; he should be as kind in his treatment of his professional brother. He thought the dentist's code of ethics should be followed. He had heard that dentists are sometimes the meanest of men in their dealings with each other. His experience was the reverse. There were those however, who would traduce any brother, no matter how skillful and learned, for the sake of making a few additional dollars. Money he thought, was proper in its place, but he must uphold the dignity of the profession. They had a broader field in the affairs of life than the mere accumulation of wealth.

Dr. Merrit, of Cleveland, made an inquiry as to the matter of bringing their business before the public, and read an advertisement which had been clipped from a paper. General theories are good he said, but we all differ in our practice, even when we think we are adhering to the code. I would like to have an expression of opinion from those present in regard to the matter.

Dr. Miller, of Elyria, said it is held that it is improper for a dentist to advertise, and that it is unprofessional. Now, if that is true, I would like to see it lived up to. No one objects to a card on the office door, to designate the man and his business. Why is not a card upon the door an advertisement? But we say, if I may advertise that much, I do not see that it would be improper to have a similar card in the newspaper, simply to tell my customers where they may find me. And so we say a professional card in the paper is in keeping with the dignity of the profession. But what is a professional card? There is very much latitude for a diversity of taste, and the consequence is we see all sizes and styles of professional cards. But if one is unprofessional, so is the other. I do not think any are. It is necessary to advertise in order io secure business. There are financial ideas to be looked after, as well as the dignity of the profession.

Dr. Buffett, of Cleveland, said the gentleman had misinterpreted the code, that it permitted a sign and a card, but did not hand bills and personal solicitations. It is a mistaken idea to send out circulars soliciting patronage. By them you say unqualifiedly you are able to do all kinds of work. Papers are full of such advertisements, and anything of that nature contains a lie. A very simple card to notify a change of room is sufficient, and will in the end prove more remunerative than a card which claims so much. If a man is worthy, people will find him out, without his flounting himself before them. It is a good plan for a young man or one changing his rooms, to have a simple card, announcing his place of business. But no reference should be made to the class of work done. A card sometimes says, anæsthetics safely administered. But physicians know they can not be safely administered in many cases, and death may frequently be traced to their door, and consumption is a prolific effect. We jeopardize ourselves and the profession by such statements. Anæsthetics may sometimes be given, but only under proper conditions of body and mind, and it requires great care to determine those conditions.

In regard to material, we should use what is best for the patient under all the circumstances. They have a right to determine what material they can afford, and we must be governed in a measure at least by them. Neither should we say anything as to prices. They are governed by the same circumstances.

In my opinion a simple card is enough, and anything more will resound to our discredit.

Dr. Horton, of Cleveland, said there were two sides of a dentist's duty. He owed a duty to the community and the community a duty to him.

But some dentists endeavor to put themselves so high they shoot way over the community, and others place themselves so low and manifest, so little faith in themselves that the community will have no confidence in them. There was only one perfect man and he does not live in these days. We are all liable to and do make mistakes, and possibly about advertising as much as anything else. Some, to be sure, consider it unprofessional for a man to state simply what he knows he can do. Is it? It is wrong for a man to say he can do that which he can not do; it is charlatanism of the worst kind, and decidedly unprofessional. It does seem as though a man might reasonably state what he knows it is possible for him to perform. Another feature of advertising calls for denunciation, that is, the habit of editors and newspaper men using the editorial "we" in advertisements for twenty-five cents a line, saying they personally know such and such things of so and so, when the truth is they know nothing about him, and simply say so because they are paid for it. The effect can not but be deleterious upon the profession at large. We all feel the evil influence in the loss of confidence produced by patronage of unqualified men puffed by such notices as that. The doctor gave numerous instances that had come under his observation. He spoke of those who come into a town or city temporarily, and drew a great deal of business by promise of cheap work. He thought the well qualified ones owed it as a duty to counteract their baneful influence by modestly calling attention to their own whereabouts. The true dentist and the community both suffer in such a case unless something of the kind is done.

Dr. Siddall, of Oberlin, said he did not think a man was at liberty to tell all of the truth in an advertisement. Suppose a man knows that on account of having no family, less expenses, etc., he can afford to do work much cheaper. So he comes out in a card and says he can do work so many per cent. cheaper than A It would not be right, it would be unjust to his professional brother. We have no right to state all that would be true in an advertisement.

Dr. Horton replied that what might be appropriate in one place might not in another. It would not be in place for a dentist where there was only one other to say he could do better work than that other. But in the cities there is a difference. There are men there who have a superior knowledge and experience as to the use of gases, and there are many others making pretensions, who have almost none. It seems to me it would be perfectly proper for him to give the fact of his knowledge and experience in an advertisement. The duties of a member to the profession receive a great deal of attention. It is hard to determine just what that duty is. There is a difference of opinion as to Christian duty. If a man has the good of the profession at heart he will never do anything that will injure a brother. Professional duty is governed by that great law of humanity, "As ye would that others should do to you, do ye so unto them." If this law is followed out, errors if any, will be those of the head, and not of the heart. We may think we are called upon sometimes to comment upon the labors of others; the only way if one has denunciation to make, is to be frank and honest in it.

TO BE CONTINUED.

